# A novel role for syndecan-3 in angiogenesis

**DOI:** 10.12688/f1000research.2-270.v1

**Published:** 2013-12-09

**Authors:** Giulia De Rossi, James R. Whiteford

**Affiliations:** 1 Centre for Microvascular Research, William Harvey Research Institute, Barts and the London School of Medicine and Dentistry, Queen Mary University of London, London, EC1 6BQ, UK

## Abstract

Syndecan-3 is one of the four members of the syndecan family of heparan sulphate proteoglycans and has been shown to interact with numerous growth factors via its heparan sulphate chains. The extracellular core proteins of syndecan-1,-2 and -4 all possess adhesion regulatory motifs and we hypothesized that syndecan-3 may also possess such characteristics. Here we show that a bacterially expressed GST fusion protein consisting of the entire mature syndecan-3 ectodomain has anti-angiogenic properties and acts via modulating endothelial cell migration. This work identifies syndecan-3 as a possible therapeutic target for anti-angiogenic therapy.

## Introduction

Angiogenesis is the process of new blood vessel formation from pre-existing vessels. This process is essential for embryonic development but it is also a feature of pathologies such as cancer and chronic inflammation (reviewed in ^1^). For angiogenesis to occur, the release of pro-angiogenic factors, which promote the transition of endothelial cells (ECs) from a quiescent to a proliferative and migratory phenotype, is required ^2– 4^. The best characterized pro-angiogenic molecules are: vascular endothelial growth factor (VEGF), fibroblast growth factor (FGF) and platelet derived growth factor (PDGF) (reviewed in ^5^). Angiogenesis represents an attractive target for the treatment of cancer, as during tumour development new blood vessels permeate the tumour mass and provide oxygen and nutrients to further enhance tumour growth. Therapies aimed at blocking angiogenesis have focused on targeting VEGF and its related receptors, however the prohibitive cost of such treatments and their side effects mean that there is still a need to discover new therapeutic anti-angiogenic targets ^4^.

Syndecans are a four member family of transmembrane adhesion receptors with diverse expression and functionality ^6, 7^. Syndecan-3 is the least well understood of the four family members. Like other syndecans, it possesses a short, highly conserved cytoplasmic domain, a single pass transmembrane domain and a large extracellular domain containing 6 glycosaminoglycan attachment sites which can be substituted by both heparan sulphate (HS) and chondroitin sulphate (CS). Syndecan-3 has the largest extracellular core protein of the syndecans and, in addition to the HS and CS modifications, the molecule possesses a number of potential sites for O-linked glycosylation resembling a mucin-rich domain which may affect both the structure and molecular interactions of syndecan-3 ^8, 9^.

Syndecan-3 Knock-Out mice, in common with other syndecan-KO animals, develop normally and only under conditions of challenge or insult are different phenotypes observed ^6^. Syndecan-3 is mainly found in the nervous system and has been shown to be a co-receptor for a number of important growth factors, including Agouti related protein (AgRP), heparin binding growth associated molecule (HB-GAM), glial cell line derived growth factor (GDNF), neurturin (NRTN), artemin (PSPN) and NOTCH, via interactions with its HS chains ^10– 14^. Interactions with AgRP, an antagonist of melanocortin, lead to altered feeding behaviours in the syndecan-3 null mouse ^14, 15^. In addition, syndecan-3 deficiency leads to the mice exhibiting more addictive behaviours to opiates such as cocaine, and this occurs as a result of its interaction with GDNF ^16^. Syndecan-3 is also expressed on satellite cells, adult skeletal muscle progenitors and has roles in the development of normal adult muscle ^13, 17– 19^.

An important emerging feature of syndecans is that sequences contained within their extracellular core domains have biological activity which can influence cell adhesion and migratory responses ^20^. Such domains have been identified in syndecan-1, 2 and -4 ^21^. The syndecans can be divided into two subfamilies based on sequence homology: syndecan-1 and -3 and syndecans -2 and -4 ^22^. Since regions of the syndecan-1 core protein are anti-angiogenic, we hypothesized that the core protein of syndecan-3 may exhibit similar properties ^23– 25^. In this study we demonstrate that the syndecan-3 extracellular core protein is able to inhibit angiogenesis by reducing the migratory potential of endothelial cells and as such may be a candidate for use in anti-angiogenic therapy.

## Materials and methods

***Cell culture:*** Brain endothelial cells (bEND3.1) and skin endothelial cells (sEND) were obtained from Health Protection Agency UK and were grown in DMEM (PAA, GE Healthcare) supplemented with 10% FBS, 2 mM L-glutamine, 1% non-essential amino acids, 1 mM sodium pyruvate and 5 μM β-mercaptoethanol (all Invitrogen), at 37°C, 10% CO _2_.

***Syndecan-3 GST fusion protein:*** The full length syndecan-3 cDNA was obtained from Source BioScience. The entire length of the mature syndecan-3 ectodomain (A ^45^-L ^380^) was amplified by PCR using the primers S3forEcoRI (ttaattgaattcgctcaacgctggcgcaatg) and S3revHindIII (ttaattaagcttctacagtatgctcttctgaggga) (Integrated DNA Technologies) and the resultant product was digested with EcoRI and HIndIII and ligated into the equivalent sites of pET41 (Novagen) according to Manufacturer’s instructions. Plasmids were verified by sequencing and transformed into the BL21 strain of *Escherichia coli* (Novagen). S3ED protein was purified from bacterial cultures which had reached an OD _600_ of 0.4 prior to the addition of 0.1 M Isopropyl β-D-1-thiogalactopyranoside (IPTG) and subsequent outgrowth for 4 hours. Affinity purification of both GST and S3ED was performed using glutathione-sepharose 4B (GE Healthcare) as described by the manufacturers.

***Aortic ring assay:*** Angiogenic sprouts were induced from rat thoracic aortas according to the method of Nicosia and Ottinetti ^26^. Briefly, aortas were dissected from cervically dislocated 180–200g 12 male Wistar rats (Harlan Laboratories) and sliced into 0.5 mm sections and incubated overnight in serum free OptiMEM (Invitrogen) at 37°C. Aortic rings were embedded in type I collagen (1 mg/ml) in E4 media (Invitrogen) containing either GST or S3ED in 48 well plates (Corning). Wells were supplemented with OptiMEM with 1% FBS and 10 ng/ml VEGF (R and D systems) and incubated at 37°C, 10% CO _2_. Angiogenic sprouts from rat aortas were counted after 4 days and 8 days respectively. Animals were housed and treated in Accordance with UK Home Office Regulations.

***EC tubule formation assay:*** Endothelial cell microtubule formation was measured as follows: ECs (5 × 10 ^4^) were seeded into 24 well plates coated with 150 µl of Matrigel (BD sciences) in the presence of either GST or S3ED. Using the Cell-IQ controlled environmental chamber (CM technologies), the plates were incubated at 37°C, 10% CO _2_ and images were captured every 15 minutes for 16 hours.

***Scratch wound migration assay:*** Confluent monolayers of bEND cells were scratched with a pipette tip, cells were then washed twice with PBS prior to the addition of growth medium supplemented with 0.5 µM of either GST or S3ED. Wounds were monitored by time lapse microscopy using an Olympus IX81 Microscope Hamamatsu Orca ER digital camera. Images were acquired every 30 minutes and subsequently analysed using Adobe Photoshop. Cell speed was quantified by manually measuring the track of individual cells migrated for 9 hours (20 cells per conditions) using Adobe Photoshop, track lengths were then divided by the time to obtain cell speed.

***Cell proliferation assay:*** EC proliferation was measured using the CellTiter 96 AQueous Cell proliferation assay kit as described by the manufacturer (Promega). bEND (5 × 10 ^3^/spot) cells were seeded in a 96-well plate (Corning) and incubated in the presence of 0.5 µM either GST or S3ED.

***Invasion assay:*** EC invasion assays through collagen matrices were performed in 24-well plates with trans-well inserts (Millipore; 8 μm pore size, polyester (PET) membrane). Membranes were coated with 10 μl of Collagen Type I mixture (Millipore; 1 mg/ml in E4 medium) containing 0.5 μM GST or S3ED. sEND cells were seeded on the gel in a homogenous single cell suspension of 5 × 10 ^3^ cells/ insert in 200 μl of DMEM + 10% FBS; 1 ml of the same medium was added to the bottom well. Invasion was measured after 6 hours after which time gels were removed with a cotton swab, the filter washed in PBS and stained with calcein (Invitrogen) and the number of cells attached to the filter was counted.

## Results

**Figure 1.**
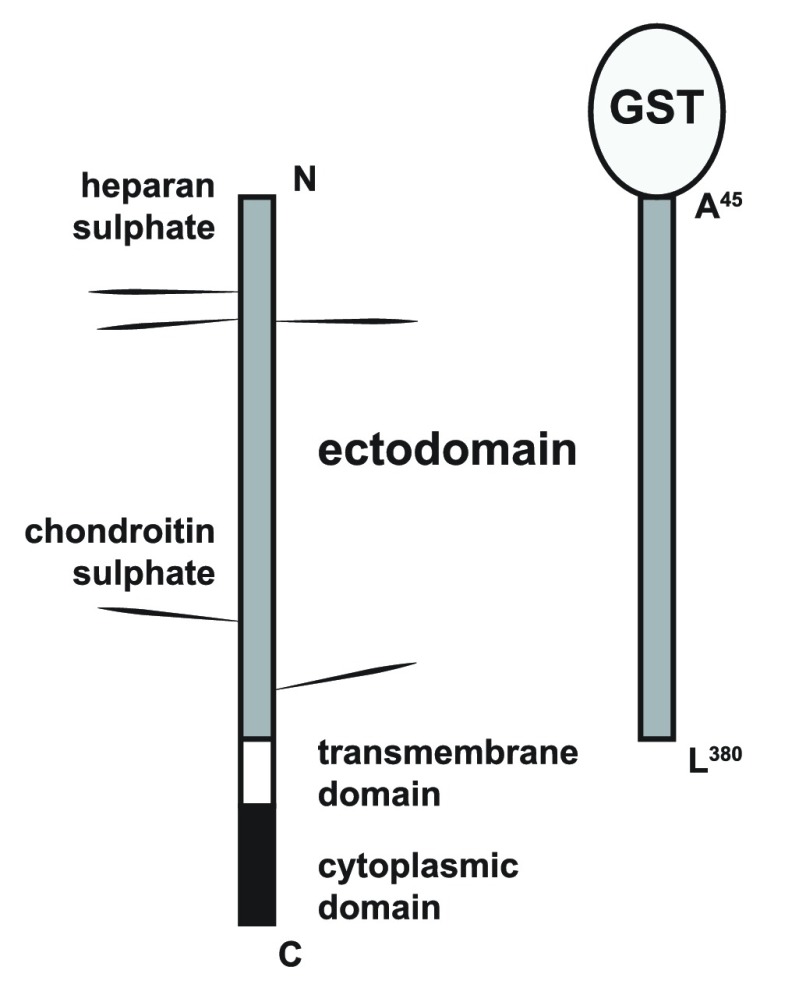
Syndecan-3 and S3ED-GST structures. Diagrammatic representation of the syndecan-3 fusion protein (S3ED) used in this study. The entire length of the syndecan-3 extracellular core protein (A ^45^-L ^380^) was fused at the N-terminus to GST.

In keeping with previous studies from our group and others in the field ^25, 27– 29^, we set out to determine whether the extracellular core protein of syndecan-3 (S3ED) had any effect on cellular responses such as cell adhesion or migration and in particular on angiogenesis. To do this we generated a fusion protein consisting of the entire length of the syndecan-3 extracellular core protein (A ^45^-L ^380^) fused at the N-terminus to GST (S3ED). S3ED was expressed and purified from bacteria and therefore was not substituted with GAGs, or *O*-linked sugars, since bacteria lack the necessary transferases to perform these post-translational modifications ( Figure 1).

**Figure 2.**
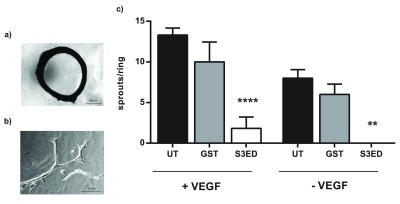
S3ED inhibits sprout formation in the rat aortic ring assay. ( **a**) Low magnification (bar=500 µm) of a rat aortic ring and ( **b**) high magnification (bar=100 µm) image of an angiogenic sprout grown under control conditions. ( **c**) S3ED inhibits angiogenic sprout formation from rat aortic rings. Rat aortic rings were seeded in Collagen I gels with 0.5 μM of either S3ED or GST in the presence or absence of VEGF. Data is the mean taken from rings from 3 different animals and error bars represent the SEM. One-way ANOVA with Bonferroni multiple comparisons was used to compare S3ED to relative UT control.

**Figure 3.**
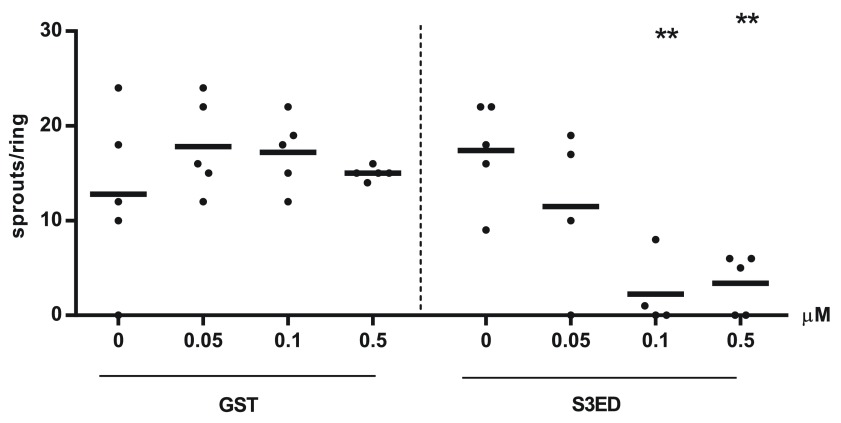
The anti-angiogenic effect of S3ED is dose dependant. Rat aortic rings were embedded in Collagen I with the indicated concentrations of either GST or S3ED in the presence of VEGF. Angiogenic sprouts were counted 4 days after seeding. Data is from 5 rings per condition. One-way ANOVA with Bonferroni multiple comparisons was used to compare S3ED 0.5 μM to S3ED 0 μM considered as a control.

In the aortic ring assay, angiogenic sprouts are formed from sections of rat aortas when embedded into a collagen I matrix ( Figure 2a and b). Sprouting occurs in the absence of VEGF although a more robust response is observed when this growth factor is included in the medium ( Figure 2c). We incorporated 0.5 µM of either GST or S3ED into the collagen I matrices to see if they affected sprout formation. Significantly fewer sprouts were observed in the presence of S3ED compared to either the untreated control or GST treated rings. This was true for both rings grown in the presence or in absence of VEGF ( Figure 2c). We then demonstrated that this inhibition of angiogenesis is dose dependent; as little as 0.5 µM of S3ED had an inhibitory effect ( Figure 3). Although 0.1 µM of S3ED also showed a statistically significant anti-angiogenic effect in this assay, we decided to use the higher dose (0.5 µM) for further experiments.

**Figure 4.**
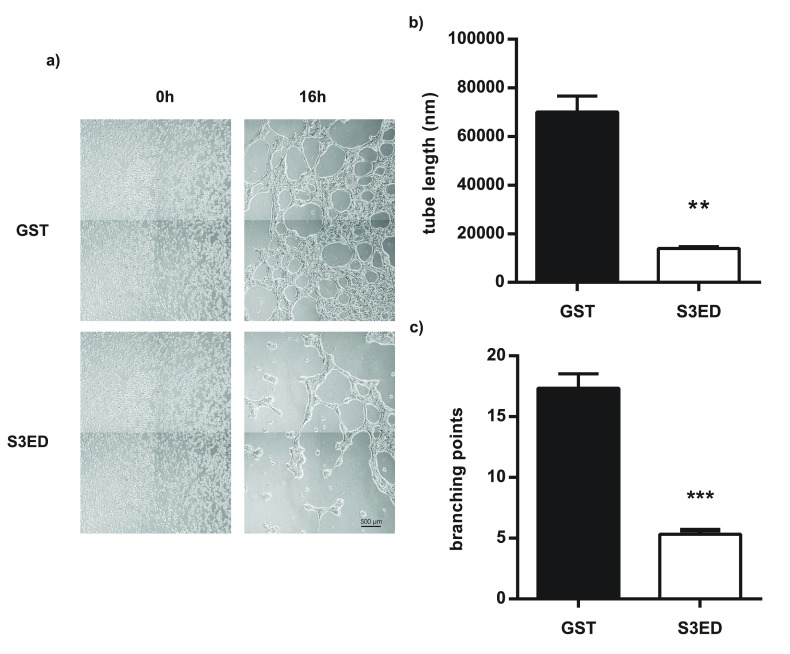
S3ED inhibits EC network formation in response to Matrigel. bEND3.1 cells were seeded on Matrigel in media containing GST or S3ED (0.5 µM) and micrographs were obtained after 16 hours ( **a**). Tube length ( **b**) and branch points ( **c**) were calculated and both were reduced in the presence of S3ED. Error bar represents SEM. T-test was used to compare S3ED to GST control.

Tubular network formation in response to matrigel is another measure of angiogenesis ^30^. Brain endothelial cells form highly branched networks when seeded on matrigel and this was unaffected when GST was added to the culture medium ( Figure 4a). In the presence of S3ED (0.5 µM) far fewer branch points were evident and the length of the microtubules was greatly reduced ( Figure 4b and c; and Movie 1 and Movie 2). Together these data suggest that S3ED has anti-angiogenic properties.

Movies 1 and 2 GST (Supp movie 1) Movie was created by assembling together phase contrast images captured using the Cell-IQ controlled environmental chamber (CM technologies) every 15 minutes for 16 hours. To obtain a broader view of the well, each picture is a collage of 4 different pictures taken on adjacent positions. Scale bar is 500 um.
S3ED (Supp movie 2) Movie was created by assembling together phase contrast images captured using the Cell-IQ controlled environmental chamber (CM technologies) every 15 minutes for 16 hours. To obtain a broader view of the well, each picture is a collage of 4 different pictures taken on adjacent positions. Scale bar is 500 um.

**Figure 5.**
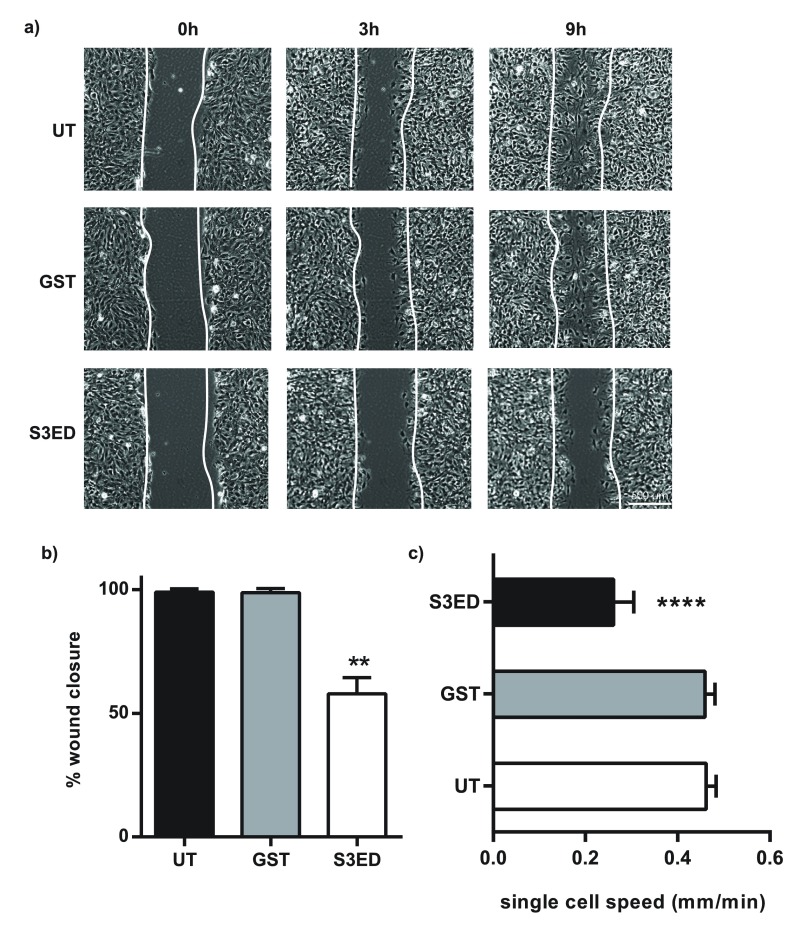
S3ED inhibits brain EC migration. Scratch wounds were made to confluent monolayers of bEND3.1 cells and micrographs were captured after 9 hours clearly showing that wound closure is reduced in the presence of S3ED 0.5 μM ( **a**). Scratch wound closure ( **b**) and single cell speeds ( **c**) were calculated. Single cell speed was calculated from 15–25 cells per condition. Error bars represent SD for ( **a**) and SED for ( **b**). One-way ANOVA with Bonferroni multiple comparisons was used to compare S3ED to relative UT control.

**Figure 6.**
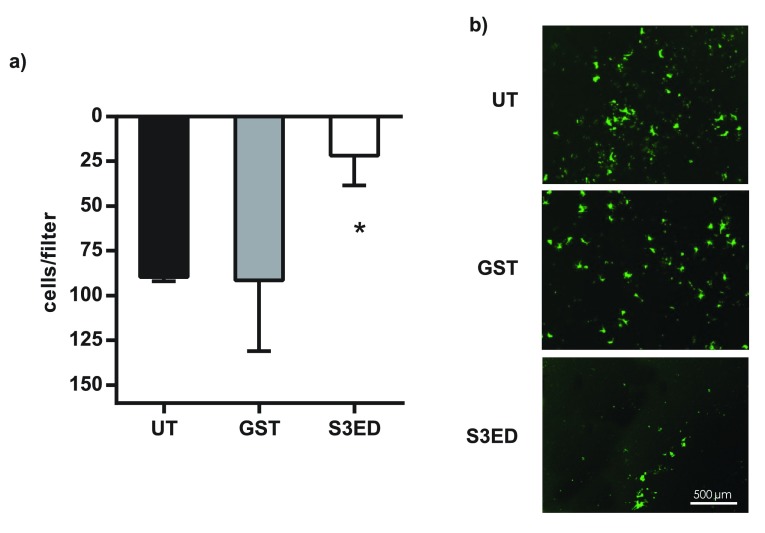
S3ED inhibits EC invasion into Collagen I. 5 × 10 ^3^ sEND cells were seeded on 10 μl of Collagen I and were allowed to migrate for 6 hours. Numbers of transmigrated cells are shown in ( **a**) and micrographs of transmigrated Calcein-labelled cells are in ( **b**). Error bars represent SD. One-way ANOVA with Bonferroni multiple comparisons was used to compare S3ED to relative UT control.

**Figure 7.**
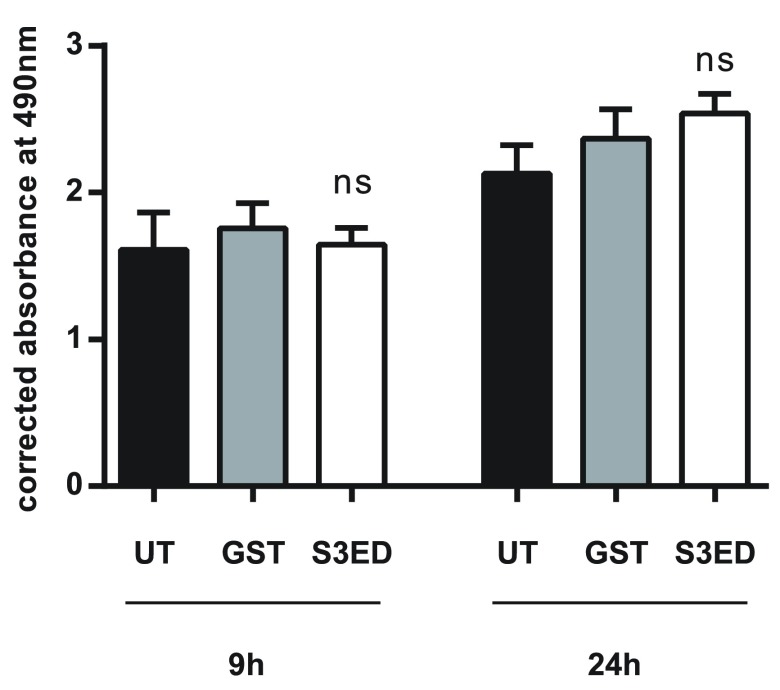
EC proliferation is unaffected by either GST or S3ED. 5 x 10 ^3^ bEND 3.1 cells were incubated in the presence of 0.5 µM of either GST or S3ED. Error bars represent SD. One-way ANOVA with Bonferroni multiple comparisons was used to compare S3ED to relative UT control showing no statistically significant difference between the two at after 9 and 24 hours.

The ectodomains of syndecan-1,-2 and -4 have all been shown to affect cell behaviour through interactions with integrins (for review see ^21^). For example, the anti-angiogenic properties of syndecan-1 have been demonstrated to occur through interactions with the αV sub-family ^25^. Integrins are the major drivers of cell migration ^31, 32^ so we set out to determine whether the inhibitory properties of S3ED were due to effects on EC migration. Scratch wound cell migration assays revealed that S3ED significantly inhibited brain endothelial cell migration as compared to GST and untreated controls ( Figure 5a). This was reflected in both percentage of wound closure and single cell speed measurements ( Figure 5b and c). We further confirmed that S3ED inhibits EC migratory potential by using a 3D culture system where we observed that S3ED, when incorporated into a collagen I matrix, inhibited endothelial cell invasion through the collagen ( Figure 6a and b). Although we demonstrate that S3ED inhibits EC migration, it could be argued that these effects may also be associated with anti-proliferative effects of this protein. To test this we performed proliferation assays on brain ECs in the presence of S3ED or GST and observed no differences in the proliferation of these cells compared with controls ( Figure 7).

Syndecan-3 raw data Fig.2 rat aortic ring assay Cells A1:F1 are column titles describing in this order: UT+VEGF, GST+VEGF, S3ED+VEGF, UT-VEGF, GST-VEGF, S3ED-VEGF. Cells below A1:F1 contains data from n=3 per condition.
Fig.3 rat aortic ring assay Cells A1:H1 are column titles describing in this order: GST 0 μM, GST 0.05 μM, GST 0.1 μM, GST 0.5 μM, S3ED 0 μM, S3ED 0.05 μM, S3ED 0.1 μM, S3ED 0.5 μM. Cells below A1:H1 contains data from n=5 per condition.
Fig.4b tube formation assay (tube length) Cells A1:B1 are column titles describing in this order: GST 0.5 μM, S3ED 0.5 μM. Cells below A1:B1 contains data from n=3 per condition.
Fig.4c tube formation assay (branching points) Cells A1:B1 are column titles describing in this order: GST 0.5 μM, S3ED 0.5 μM. Cells below A1:B1 contains data from n=3 per condition.
Fig.5b scratch wound assay (wound closure) Cells A1:C1 are column titles describing in this order: UT, GST 0.5 μM, S3ED 0.5 μM. Cells below A1:C1 contains data from n=3 per condition.
Fig.5c scratch wound assay (single cell speed) Cells A1:C1 are column titles describing in this order: UT, GST 0.5 μM, S3ED 0.5 μM. Cells below A1:C1 contains data from n=15-15 per condition.
Fig.6 invasion assay Cells A1:C1 are column titles describing in this order: UT, GST 0.5 μM, S3ED 0.5 μM. Cells below A1:C1 contains data from n=4 per condition.
Fig.7 proliferation assay Cells A1:F1 are column titles describing in this order: UT 9h, GST 9h, S3ED 9h, UT 24h, GST 24h, S3ED 24h. Cells below A1:C1 contains data from n=4 per condition.

## Discussion

Here we show for the first time that the ectodomain of syndecan-3, like the other syndecan family members, has cell adhesion regulatory properties and acts as an inhibitor of angiogenesis. This strongly suggests that adhesion regulatory domains in syndecan extracellular core domains exist in all four family members and also provides further insight as to how these molecules function. Interestingly, there is very little sequence homology between syndecan ectodomains ^32^, and the adhesion regulatory motifs identified in syndecan-1,-2 and -4 are not present in S3ED. In addition, syndecan-3 is unique amongst the family since its core protein possesses a mucin-rich domain and it is likely that these *O*-linked sugars will also have a role in its molecular interactions. Although syndecan-3 expression is mostly associated with cells of the nervous system, it has also been found to be associated with cells related to maternal and foetal circulation ^33^. Vascular defects in the syndecan-3 null mouse have not been reported but the identification of S3ED as a regulator of angiogenesis suggests that this may be a fertile area for future research. This work identifies syndecan-3 as a potential therapeutic target in pathologies where angiogenesis is a feature.
