# Supplementary material for: A novel role for syndecan-3 in angiogenesis
Source: F1000Res. 2013 Dec 9;2:270. [Version 1] doi: 10.12688/f1000research.2-270.v1 (PMC3886797; doi:10.12688/f1000research.2-270.v1)
Supplement: Peer review history

# Peer review: Referee response for version 1

**Published:** 2014-01-07

**Authors:** Jonas Jacobsen

**Affiliations:** 1 Enzymes, Chr. Hansen A/S, Hørsholm, Denmark

The work described by researchers Whiteford and De Rossi is placed in the scientific field angiogenesis. The research group has identified the mature syndecan-3 ectodomain in fusion with a GST protein as a possible therapeutic target for anti-angiogenic therapy. A therapy that could prove relevant in the treatment of cancer.

The biological activities of extracellular core domain of Syndecan-3 is both novel and interesting and deserve publication.

The only minor point of criticism to the authors is the following:

In the discussion it is mentioned that O-linked glycosylation in the mucin-rich region of Syndecan-3 may also engage in molecular interactions. While this may be true it is also a fact that the expressed syndecan-3 fusion protein is not glycosylated because in was produced in the BL21 strain of *Escherichia coli*.

I have read this submission. I believe that I have an appropriate level of expertise to confirm that it is of an acceptable scientific standard.

# Peer review: Referee response for version 1

**Published:** 2013-12-20

**Authors:** Mark Morgan

**Affiliations:** 1 Institute of Translational Medicine, University of Liverpool, Liverpool, UK

This is an interesting study highlighting the potential role of syndecan-3 in the regulation of angiogenesis. The study was well executed and the manuscript well written.

 

The authors show that recombinant syndecan-3 ectodomain (S3ED) inhibits angiogenic sprouting in a dose-dependent manner, using *ex vivo* aortic ring assays. They further show that S3ED inhibits endothelial cell migration and suggest that this is responsible for its anti-angiogenic effect.

 

This work warrants publication as it raises many new questions (beyond the realms of this project), about the non-neuronal roles of a relatively understudied syndecan. For example:

- What are the effects of S3ED *in vivo* and can syndecan-3 be targeted therapeutically?
- Does endogenous syndecan-3 (either shed or membrane-bound) serve a role for fine-tuning angiogenic processes *in vivo*?
- What is the specific mechanism by which S3ED inhibits endothelial cell migration and angiogenesis? Does it involve modulation of integrin and/or growth factor receptor function?

No doubt these questions will form the basis of many future studies.

 

Minor Points:

1. In Fig 2 it would be useful to include a representative image of an S3ED-treated aortic ring. Are angiogenic sprouts completely absent or are they just stumps?
   
2. The Transwell "invasion" assays described use a very thin coating (10ul) of non-crosslinked collagen type 1 and a relatively short time period (6Hrs). Consequently, they are not a good read-out of "invasion" *per se*, but rather of a mode of migration. Therefore the process should be described as "migration through collagen", or perhaps "invasive migration".

I have read this submission. I believe that I have an appropriate level of expertise to confirm that it is of an acceptable scientific standard.

# Peer review: Referee response for version 1

**Published:** 2013-12-10

**Authors:** Alberto Grossi

**Affiliations:** 1 Department of Food Science and Food Microbiology, Copenhagen University, Copenhagen, Denmark

This is a well conducted study which highlights an unforeseen role for the heparan sulphate proteoglycan syndecan-3. Additionally, beyond the realms of this study, these data suggest more detailed knowledge of the syndecan-3 expression profile is required.

I have read this submission. I believe that I have an appropriate level of expertise to confirm that it is of an acceptable scientific standard.
